# Study on the risk of soil heavy metal pollution in typical developed cities in eastern China

**DOI:** 10.1038/s41598-022-07864-3

**Published:** 2022-03-09

**Authors:** Yan Li, Zhen Dong, Dike Feng, Xiaomian Zhang, Zhenyi Jia, Qingbin Fan, Ke Liu

**Affiliations:** 1 Collaborative Innovation Center of Sustainable Forestry, Nanjing Forestry University, Nanjing, Jiangsu China; 2 Key Laboratory of Watershed Geographic Sciences, Nanjing Institute of Geography and Limnology, Chinese Academy of Sciences, Nanjing, Jiangsu China; 3 Key Laboratory of Geographic Information Science of the Ministry of Education, School of Geographic Sciences, East China Normal University, Shanghai, China; 4 Zhejiang Academy of Forestry Sciences, Hangzhou, Zhejiang China; 5 Key Laboratory of Desert and Desertification, Northwest Institute of Eco-Environment and Resources, Chinese Academy of Sciences, Lanzhou, 730000 China; 6 School of Geography and Ocean Science, Nanjing University, 163 Xianlin Road, Nanjing, Jiangsu China

**Keywords:** Ecology, Environmental sciences

## Abstract

Enrichment of heavy metals in urban soils has become a major regional environmental risk. At present, research on the soil heavy metals in cities lacks risk spatial correlation analyses between different heavy metals, and there is a relative lack of assessments of the ecological and health risks. We selected Wuxi, a typical developed city of eastern China, collected and tested the contents of heavy metals in the urban soils of Wuxi in May 2020. Combined with Pb isotope analysis, ecological and health risk assessment, we found that the high heavy metal concentrations in Wuxi are mainly located in the central and western regions, and that the changes in spatial fluctuation are relatively small. The Pb isotopes in the urban soils of Wuxi are distributed in areas, such as those are related to coal combustion, automobile exhaust and urban garbage, indicating that the heavy metals in the urban soils of Wuxi are affected by human activities such as coal combustion and automobile exhaust. The average value of the potential ecological risk index of soil heavy metal Cd is 80.3 (the threshold: 40), which represents a high-risk state. Whether adults or children, the risk of soil heavy metals via ingestion is much higher than that through skin exposure. High health risk values are present in the central area of Wuxi and decrease in a ring-shaped pattern, which is similar to the population distribution of Wuxi and greatly increases the potential risk from soil heavy metals, which should be given close attention. We should develop and use clean energy to replace petroleum fossil fuels, especially in densely populated areas. This study provides technical support for the prevention and control of urban heavy metal pollution.

## Introduction

With the development of industrial economy, heavy metals in the environment are accumulating day by day. Heavy metal elements (such as cadmium (Cd), cobalt (Co), chromium (Cr), copper (Cu), manganese (Mn), nickel (Ni), lead (Pb), zinc (Zn)) pose serious threats to the activities and lives of organisms only when they exceed a certain limit in soil^1,2^. Some elements (such as Cd, Cr and Pb), even in trace amounts, have essential harmful effects on organisms^3,4^. These elements do not play a key role in the growth and development of organisms. Heavy metals accumulations in the human body at certain levels will cause changes in various physiological functions of the human body and will eventually show the three pathogenic effects of "carcinogenesis", "teratogenesis" and "mutagenicity"^5,6^. It is becoming increasingly important to examine the ecological risks that are caused by heavy metals to the ecological environment and the health risks to the human body.

As places where heavy metals collect, soils play an important role in the migration and transformation of heavy metals. Human activities have an important impact on the accumulation of heavy metals in soils^7,8^. The manufacturing and processing of factory operations and automobile exhaust emissions Pb to the enrichment a of large number of heavy metals in soil^9^. The high intensity of human activities in cities will lead to the enrichment of more heavy metals and other pollutants in the environment. More importantly, the populations are generally concentrated in cities but are relatively lower in the suburbs and villages, which leads to greater human health risks in urban central areas. Therefore, it is more urgent to explore the risk of heavy metal pollution in soil of developed cities^10,11^. Wuxi is one of the central cities in the Yangtze River Delta that is approved by the State Council and is a typical developed city in eastern China. There are studies on heavy metals in Wuxi urban soil^11,12^, but these researchs occurred nearly ten years ago. There is a lack of recent data on soil heavy metal concentration and pollution risk in Wuxi. With dense population, developed industry and numerous factories, Wuxi is a typical developed city in eastern China. The characteristics and level of heavy metal pollution here can reflect the pollution status of other developed cities to a certain extent. Research on the risk of heavy metals in the urban soils of Wuxi has become particularly urgent and important.

Pb isotopes are a good indicator of the sources of heavy metal pollution, can effectively distinguish the sources of heavy metals in soil, and provide a prerequisite to further explore the risks of heavy metals that are caused by various pollution sources^13–18^. However, in the studies of heavy metals in urban soils, there are few studies on Pb isotopes and relevant research results are lacking. In addition, the existing soil heavy metal risk studies mainly focus on spatial analyses of a single heavy metal^19^, while the spatial correlation characteristics of multiple heavy metal risks are seriously lacking, and there is also a lack of relevant analysis methods.

This study systematically researched the ecological risk and health risk of heavy metals in Wuxi city soils. Different from previous studies, this study combined with lead isotope study discussed the different sources of heavy metals in Wuxi urban soil, and put forward pollution control measures. The main research purposes are as follows: (1) explore the present spatial distribution characteristics of heavy metals in the urban soils of Wuxi, (2) analyse the Pb isotope characteristics of heavy metals and the characteristics of human health risks, and (3) clarify the spatial distributions and spatial correlations of the ecological risks of different heavy metals. The results of this study will provide guidance and reference for risk-source analysis, early warning and management of soil heavy metals in developed cities.

## Sample collection and testing

### Sample collection and experimental treatment

**Figure 1:**
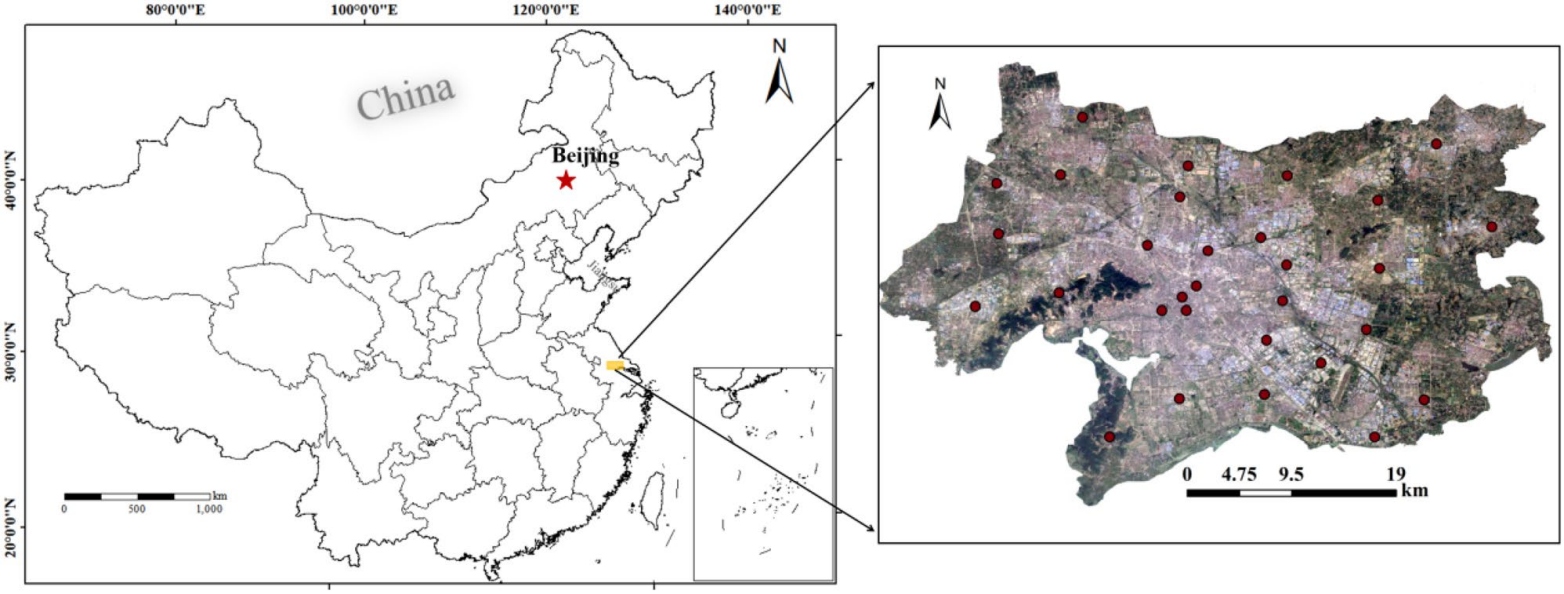
Location of the study area and distribution of sampling points [the figure was generated by Yan Li using the ArcGIS 10.2 (http:// https://developers.arcgis.com/)].

The urban soil sampling points in Wuxi are mainly distributed in the urban area of Wuxi and exclude the secondary areas of Wuxi, such as Yixing City and Jiangyin City. The sampling points cover the entire urban area of Wuxi (Fig. 1). The distribution density of sampling points is relatively high in the centre of the urban area and relatively low in the suburbs. The thickness of the soil samples that were collected at each sampling point were 0–5 cm, and a total of 30 soil samples were collected. After the soil samples were brought to the laboratory, they were dried by natural air to remove gravel, fallen leaves and other sundry materials. The air-dried soil samples were ground, passed through a 100 mesh sieve and stored in self-sealing bags.

Approximately 0.1 g of the ground soil samples were weighed into a 25-ml Teflon beaker, and the soil samples were digested by four acid digestion methods (e.g., concentrated hydrochloric acid, concentrated nitric acid, perchloric acid and hydrofluoric acid). The pure water used in the digestion process was Milli-Q ultrapure water, and the temperature of the asbestos mesh heating plate was approximately 200 °C. To improve the accuracy of the element content tests, the reference material GBW 07,405 was tested after every five samples for corrections. The heavy metals Mn, Zn, Cr, Ni, Pb, Cu, Co and Be were tested by inductively coupled plasma atomic emission spectrometry (ICP-AES, Optima5300DV, PerkinElmer, USA), and the heavy metal Cd was tested by inductively coupled plasma atomic emission spectrometer (ICP-MS, PerkineElmer SCIEX, Elan 9000). The specific operational steps are shown in the supplementary materials. Based on the Pb concentrations that were determined by ICP-AES, the extracts were diluted to obtain sample solutions with concentrations of approximately 30 μg·L^-1^ that were then tested using ICP-MS. The accuracy and precision of the Pb isotope determinations were controlled by the Pb isotope reference material SRM 981 (National Bureau of Standards). To correct the accuracy of the Pb isotope testing, the Pb isotope reference material SRM 981 was tested after every two samples^20^.

### Analysis method

This paper uses Microsoft Excel 2019 (Redmond, Washington, USA) to perform basic data calculations; IBM SPSS 22.0 (IBM Corporation, Armonk, New York, USA) software was used for data description, statistics and correlation analysis; OriginPro 9.1 (OriginLab, Northampton, Massachusetts, USA) was used to draw some of the graphs; ArcGIS 10.2 (ESRI Corporation Redlands, California, USA) was used to complete the spatial data analyses and spatial mapping. GIS techniques are widely used to qualitatively identify pollution sources. They can transform the very large amounts of abstract data into intuitive spatial distribution maps. GIS spatial analyses can generally distinguish point source pollution from nonpoint source pollution. The spatial data interpolation method that was selected for use in this study was the inverse distance spatial interpolation (IDW) in the GIS spatial analysis function.

1. Potential ecological risk index.

In this study, the Hakanson^21^ potential ecological risk index method was adopted to evaluate the risk degrees of heavy metals in the soils of Wuxi^21^. This method combines ecology, biochemistry and other aspects to evaluate the potential ecological risk from heavy metals. The formula is as follows:

$${\text{C}}_{{\text{f}}}^{{\text{i}}} = \frac{{{\text{C}}^{{\text{i}}} }}{{{\text{C}}_{{\text{n}}}^{{\text{i}}} }}$$

$${\text{E}}_{{\text{r}}}^{{\text{i}}} = {\text{T}}_{{\text{r}}}^{{\text{i}}} \times {\text{C}}_{{\text{f}}}^{{\text{i}}}$$

where ${\text{ C}}_{{\text{f}}}^{{\text{i}}}$ is the pollution coefficient of a single heavy metal,${\text{ C}}^{{\text{i}}}$ is the content of heavy metals in the measured experimental samples, and ${\text{C}}_{{\text{n }}}^{{\text{i}}}$ is the reference ratio for heavy metal pollutant i. In this study, the Wuxi soil background value was used as the reference ratio,${\text{ T}}_{{\text{r}}}^{{\text{i}}} { }$ was the corresponding toxicity coefficient of pollutant i, and the toxicity coefficients of each heavy metal were Cd = 30, Cu = Ni = Pb = 5, Cr = 2, and Ti = Zn = Mn = 1^21,22^. ${\text{E}}_{{\text{r}}}^{{\text{i}}} { }$ is the potential ecological risk index of a single heavy metal element i.

(2) Human health risk assessment.

After heavy metals enter the human body, they will cause functional disorders and irreversible damage and pose specific threats to human health. Thus, the health risk of pollutants to the human body in environmental media is a problem that scholars at home and abroad pay significant attention to. By using the health risk assessment model that is recommended by the US EPA and by referring to the research results of the domestic exposure route parameter settings, this paper modifies some parameters of the pollutant exposure model and discusses the health risks that are caused by multichannel exposure to heavy metals in children and adults in the study area^23^. The ways that soil heavy metals can enter the human body include ingestion and skin contact. The detailed calculation process is provided in the supplementary materials.

(3) Bivariate LISA analysis

The local Moran’s I statistic is often used to analyse chemical element hotspots in the environment and is a well-accepted indicator for bivariate LISA analysis^24,25^. The method that was used to calculate the bivariate local Moran’s I (*M*_*kl*_) statistic is as follows:

$$M_{kl} = Z_{k}^{i} \sum\limits_{j = 1}^{n} {W_{ij} } Z_{i}^{j} ,$$

where $Z_{{\text{l}}}^{{\text{i}}} = [X_{l}^{i} - \mathop X\limits^{ - } K]/\partial l$, $Z_{l}^{{\text{i}}} = [X_{l}^{j} - \mathop X\limits^{ - } l]/\partial l$; $X_{k}^{{\text{i}}}$ is the value of variable *k* at point *i*; $X_{l}^{j}$ is the value of variable *l* at point *j*; $\mathop X\limits^{{\text{\_}}} K$ and $\mathop X\limits^{\_} l$ are the average values of variables *k* and *l*, respectively; $\partial k$ and $\partial l$ are the variances of variables *k* and *l*, respectively; and $W_{ij}$ is the spatial weight matrix, which can be represented based on a distance weighting between locations *i* and *j*. In this study, we used Geoda (http://geodacentre.github.io/) to calculate the bivariate Moran’s I statistic.

According to the local Moran’s I values of all samples, we were able to use the corresponding statistical significances and local Moran’s I values to cluster the features. In the results, “high” and “low” indicate that the pollutant concentrations are higher or lower, respectively, than those at the surrounding sites. Based on Moran’s I, the studied region can be divided into four categories for two classes of pollutants: a high–high value cluster, high–low value cluster, low–high value cluster, and low–low value cluster^26,27^. In addition, another possibility is that the local Moran’s I statistic cannot compare the concentrations in subregions, which introduces a fifth category: a nonsignificant cluster^26,27^.

### Ethical approval

All authors have read and approved this version of the article.

## Results and discussion

### Characteristics of heavy metal concentrations

On the basis of the soil sample collection and chemical analysis, the concentration data for heavy metals in the urban soils of Wuxi were obtained. Through the statistical analysis of the soil heavy metal concentration data (Table 1), on the whole, the concentration of each heavy metal is as follows: Mn > Zn > Cr > Ni > Pb > Cu > Co > Be > Cd. Among these, the concentration range of Cr was 64.5–99 mg kg^-1^, and the average concentration was 72.9 mg kg^−1^. The concentration range of Ni was 31.4–67.5 mg kg^−1^, and the average concentration was 38.2 mg kg^−1^. The concentration range of Cu was 19.8–37.2 mg kg^−1^, and the average concentration was 25.5 mg kg^−1^. The concentration range of Zn was 72.4–1146 mg kg^−1^, and the average concentration was 90.2 mg kg^−1^. The concentration range of Cd was 0.34–1.06 mg kg^−1^, and the average concentration was 0.51 mg kg^−1^. The concentration range of Pb was 25.6–66.4 mg kg^−1^, and the average concentration was 37.6 mg kg^−1^. The variation coefficients of urban soil heavy metal concentration in Wuxi is between 0.09 and 0.33, which is less than 1. The spatial fluctuation of urban soil heavy metal concentration in Wuxi is small, indicating that the sources may be the same or similar.

**Table 1:** Statistics of the heavy metal concentrations and Pb isotope ratios in the urban soils of Wuxi city (unit of heavy metal: mg kg^−1^; CV: coefficient of variation).

Element	Min	Max	Mean	Standard deviation	Variance	CV
Be	1.5	2.6	1.95	0.26	0.07	0.13
Cr	64.5	99	72.9	6.8	45.8	0.09
Mn	629	1210	851.8	139.4	19,431.5	0.16
Co	12.2	17.9	14.2	1.2	1.4	0.08
Ni	31.4	67.5	38.2	7.2	51.3	0.19
Cu	19.8	37.2	25.5	3.3	11.0	0.13
Zn	72.4	146	90.2	13.5	183.5	0.15
Cd	0.34	1.06	0.51	0.17	0.03	0.33
Pb	25.6	66.4	37.6	9.0	80.9	0.24
^208^Pb/^206^Pb	2.09	2.12	2.10	0.0068	0.00005	0.003
^206^Pb/^207^Pb	1.17	1.18	1.177	0.0046	0.00002	0.004

**Figure 2:**
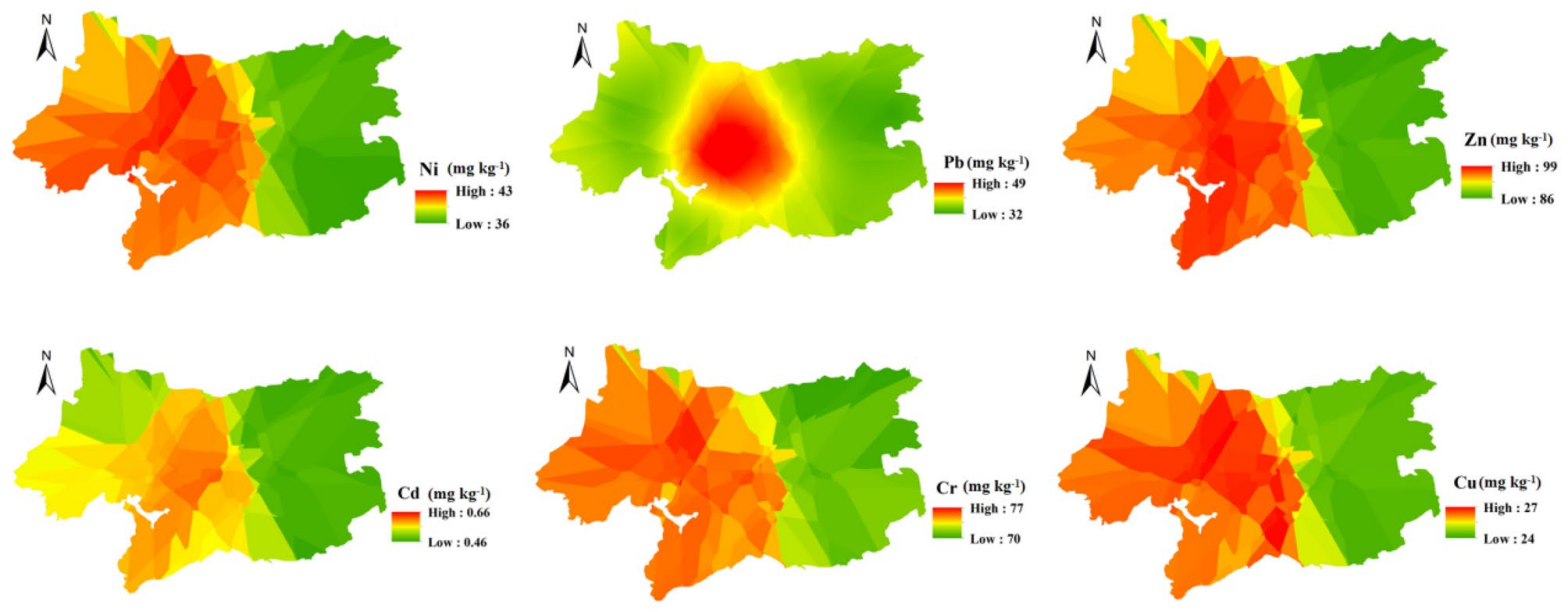
Spatial distribution characteristics of heavy metals in the urban soils of Wuxi city (unit: mg kg^−1^) [the figure was generated by Yan Li using the ArcGIS 10.2 (http:// https://developers.arcgis.com/)].

By analysing the spatial distributions of the urban soil heavy metal concentrations in Wuxi, several obvious spatial distribution characteristics are found (Fig. 2). First, the heavy metals have high values in the central area of Wuxi, due to where has a high population density and various industries. The central aggregation of Pb is more obvious. Due to the dense roads in the city centre, vehicle traffic, bus stop signs and gas stations are mostly concentrated here, which will lead to Pb contents in this area that are significantly higher than those in other areas. In addition to the heavy metal concentrations, such as those for Cu, Zn and Cr in the downtown area, there are also areas with high values in western Wuxi and low values in eastern Wuxi. This phenomenon may be related to the land use types in Wuxi. In the western area of Wuxi, most land use types are urban and construction land, and the soils in this area are greatly disturbed by human activities. In the eastern region of Wuxi, woodland and grassland account for a large proportion of the land use types, which are less disturbed by human activities.

### Source analysis of heavy metals

Exploring for heavy metal pollution from emission sources is an important prerequisite for the study of urban soil pollution. By analysing the sources of heavy metals in soil environments, we can accurately determine which industries are major sources^28–30^ and whether there is homologous pollution. This is not only a theoretical basis for the study of lake sediment pollution and to clarify the risks brought by different pollution sources to the urban soil environment but also provides important guides for local government control of specific polluting industries and pollutant emissions. Based on this, the correlations and significance of heavy metals in the urban soils of Wuxi were analysed (Table 2). Generally, a heavy metal pollution source will emit multiple heavy metals at the same time. If the pollution source has a large emission, the concentration of these heavy metals in the environment will show a high level; on the contrary, if the emission of this pollution source is small, the concentration of these heavy metals in the environment will show a low level^10^. The correlations between the heavy metals Zn, Cr, Ni, Pb, Cu and Cd are between 0.655–0.907 and show strong correlations and significance at a level of 0.01. The strong significant correlations between different heavy metals indicate that these heavy metals have similar emission sources and transmission routes, which also means that they have consistent sources.

**Table 2:** Correlations of Heavy Metals in the Urban Soils of Wuxi City.

	Cr	Mn	Co	Ni	Cu	Zn	Cd	Pb	Be
Cr	1	.499**	.822**	.897**	.867**	.892**	.625**	.627**	− 0.028
Mn	.499**	1	0.316	0.36	.432*	.570**	.468**	0.357	− 0.044
Co	.822**	0.316	1	.630**	.656**	.591**	.422*	.376*	− 0.013
Ni	.897**	0.36	.630**	1	.893**	.903**	.664**	.693**	− 0.08
Cu	.867**	.432*	.656**	.893**	1	.907**	.716**	.696**	− 0.071
Zn	.892**	.570**	.591**	.903**	.907**	1	.830**	.753**	− 0.086
Cd	.625**	.468**	.422*	.664**	.716**	.830**	1	.655**	− 0.201
Pb	.627**	0.357	.376*	.693**	.696**	.753**	.655**	1	0.01
Be	− 0.028	− 0.044	− 0.013	− 0.08	− 0.071	− 0.086	− 0.201	0.01	1

**There was a significant correlation at the 0.01 level (bilateral). * There was a significant correlation at the 0.05 level (bilateral).

**Figure 3:**
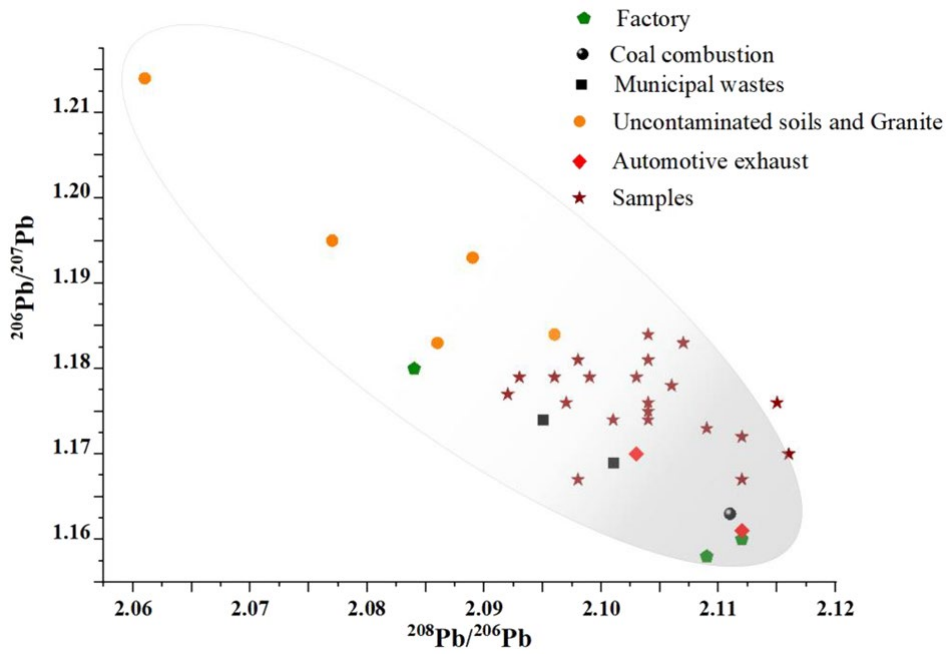
Comparison of the Pb isotope compositions in the urban soils of Wuxi city with known sources.

To further determine which industries are the sources of the heavy metals found in the urban soil of Wuxi, we analysed the Pb isotope data. The variation range of ^208^Pb/^206^Pb in soil is 2.09–2.12, and the average value is 2.10. The variation range of ^206^Pb/^207^Pb in soil is 1.17–1.18, and the average value is 1.177 (Table 1). After consulting relevant literature and materials, the main pollution sources of heavy metals in cities in eastern China include coal combustion, oil combustion, factory emissions, municipal wastes and so on^3^. Therefore, we collected the corresponding Pb isotope data in the emissions of heavy metal pollution sources. By collecting and comparatively analysing the Pb isotope data of known pollution sources (Fig. 3), it was determined that the Pb isotopes of the urban soil heavy metals in the soils of Wuxi have distinct characteristics. First, the Pb isotope distributions in the soils of Wuxi are relatively concentrated, and the ranges of variation are relatively small, which indicate that these heavy metals may have the same source or similar sets of sources. Second, the Pb isotopes in the urban soils of Wuxi city have few similarities with those of the uncontaminated soils and granites in eastern China; in contrast, the Pb isotopes in the urban soils of Wuxi are distributed in areas that are associated with coal combustion, automobile exhaust and urban waste (supplementary materials). The urban soil heavy metals in Wuxi generally have similar pollution sources and are greatly affected by human activities such as coal combustion and automobile exhaust emissions. Wuxi has a developed industrial economy and large numbers of factories. In the production and processing activities, the combustion of energy and fuel and the incomplete utilization of raw materials will lead to the enrichment of pollutants in the surrounding environment. By comparing other studies^30,31^, the Pb isotope analysis results in this study well indicate the source of soil heavy metals in Wuxi and make up for the Pb isotope data in this area. In the process of urban development, we should develop and apply clean energy, reduce the utilization of petroleum fossil fuels, and control the enrichment of heavy metals and other pollutants in the soil from the source.

### Ecological risk analysis

**Figure 4:**
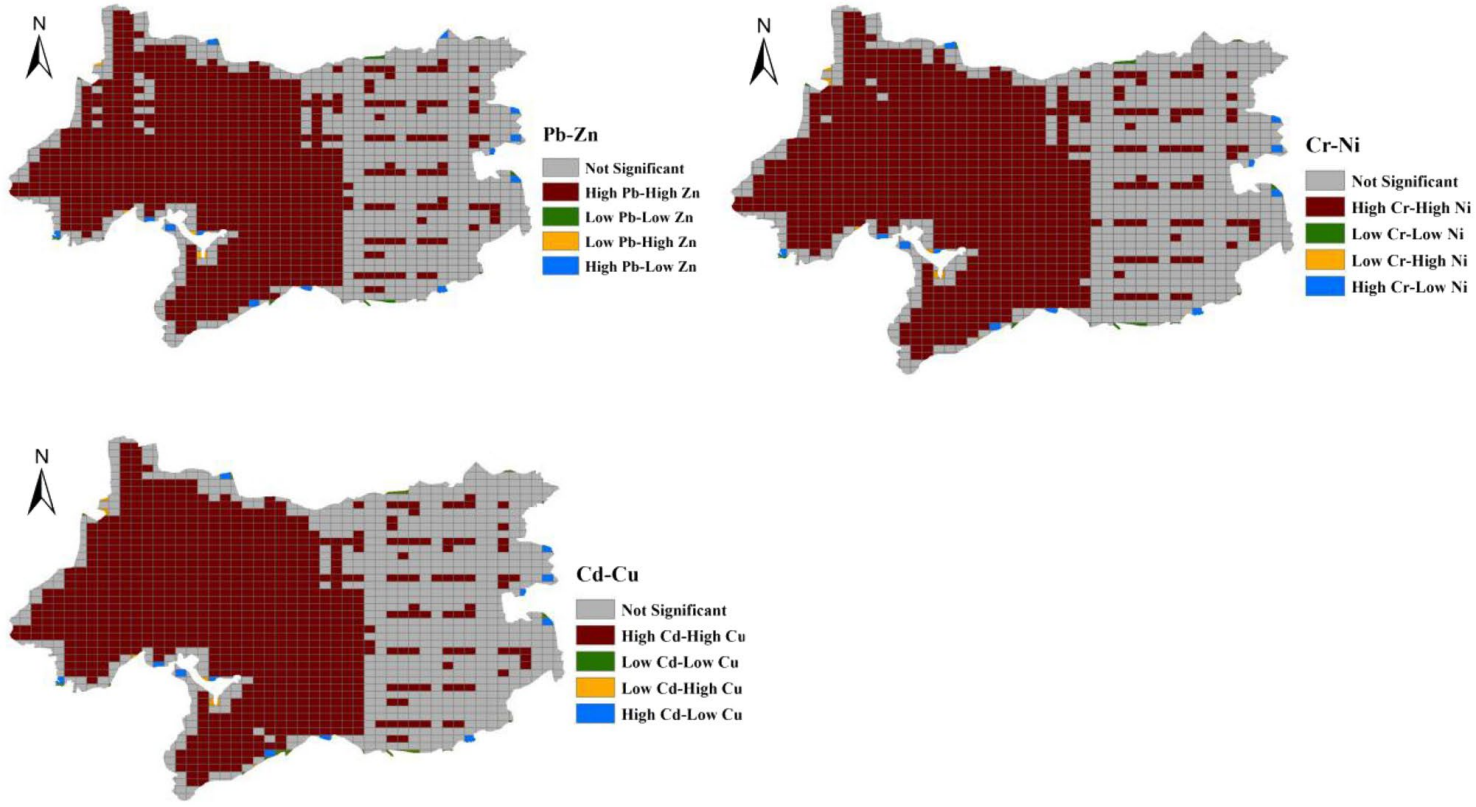
LISA analysis of the ecological risks from different heavy metals [the figure was generated by Yan Li using the ArcGIS 10.2 (http:// https://developers.arcgis.com/)].

By calculating the potential ecological risk index for the heavy metals in the urban soils of Wuxi, the risks of heavy metals in the Wuxi soils were evaluated (Table 3). According to previous studies^21^, an E_i_ value lower than 40 indicates that a heavy metal is in a low-risk state at this location, and E_i_ values greater than or equal to 40 indicate that a heavy metal represents a high-risk state at this location. The average value of the potential ecological risk index of soil heavy metal Cd in Wuxi is 80.3, which represents a high-risk state. The average distributions of the potential ecological risk indexes of the heavy metals Cr, Cu, Zn, Pb and Ni are 1.8, 4.3, 1.1, 5.5 and 4.8, respectively, which all indicate a low-risk state. The risk statuses of different heavy metals may show certain correlations in space, which may be mutually complementary or antagonistic. Examining the spatial interactions of different heavy metal compound pollutants in urban soils plays an important role in the prevention and control of urban heavy metal pollution. Based on this, we used the Lisa analysis method to explore the spatial correlations of the different heavy metal risks in the urban soils of Wuxi (Fig. 4). The Moran scatter diagram can be divided into four quadrants that correspond to four different spatial patterns. High means that the variable value is higher than the average value, and Low means that the variable value is lower than the average value. In the upper right quadrant (High–High), a high-value area is surrounded by high-value neighbours; in the upper left quadrant (Low–High), a low-value area is surrounded by high-value neighbours; in the lower left quadrant (LL), a low-value area is surrounded by low-value neighbours; and in the lower right quadrant (High–Low), a high-value area is surrounded by low-value neighbours. High-High and Low-Low indicate that the differences between the region and its surrounding areas are small; that is, the regions with higher or lower values are concentrated, while the Low–High and High–Low quadrants indicate that the variable values between a region and its surrounding areas are different to a certain extent.

**Table 3:** Ecological risk and health risk analysis of heavy metals in the urban soils of Wuxi (Cr-E represents the ecological risk of metal element Cr; Ni-E represents the ecological risk of metal element Ni; Cu-E represents the ecological risk of metal element Cu; Zn-E represents the ecological risk of metal element Zn; Cd-E represents the ecological risk of metal element Cd; Pb-E represents the ecological risk of metal element Pb; ADDderm-C is the average exposure to skin contact pathways for child; ADDderm-A is the average exposure to skin contact pathways for adult; ADDing-C is the average daily exposure to intake pathway for child; ADDing-A is the average daily exposure to intake pathway for adult; HI-C is the total health risk caused by accumulation of heavy metals in multiple ways in the same environmental medium for child; HI-A is the total health risk caused by accumulation of heavy metals in multiple ways in the same environmental medium for adult).

Index	Min	Mix	Mean	Standard deviation	Variance
Cr-E	1.6	2.4	1.8	0.17	0.03
Ni-E	3.9	8.4	4.8	0.89	0.79
Cu-E	3.4	6.3	4.3	0.56	0.32
Zn-E	0.9	1.8	1.1	0.17	0.03
Cd-E	53.8	166.9	80.3	26.4	695.3
Pb-E	3.7	9.7	5.5	1.3	1.7
ADDderm-C	0.01	0.01	0.008	0.0008	
ADDderm-A	0.001	0.002	0.001	0.0001	
ADDing-C	0.05	0.12	0.07	0.0151	
ADDing-A	0.01	0.03	0.016	0.0036	
HI-C	0.06	0.13	0.078	0.0157	
HI-A	0.01	0.03	0.018	0.0037

In this study, two main results were obtained from spatial correlation Lisa analysis between different heavy metals. One is a High-High area, which is mainly distributed in the central and western regions of Wuxi city, which is consistent with the spatial distribution of the urban soil heavy metal concentrations in Wuxi city and is strongly disturbed by human activities. The other is the insignificant area, in which there are also large numbers of factories and enterprises and in which the forestland and grassland are distributed at intervals, which leads to an insignificant spatial correlation of soil heavy metal contents. Based on the above analysis, the high-risk areas for heavy metals in the urban soils of Wuxi are mainly concentrated in the central and western regions, and the relevant management activities need to be given great attention. In the eastern region, sporadic high-risk areas are also present, which should also receive due attention. Moran's I is a method to measure the interdependence and degree of objects or phenomena by constructing statistics on certain characteristics or attributes for a certain spatial unit in the study area and the surrounding spatial units. It can be used to describe the spatial characteristics of spatial units such as aggregation or outliers in the distribution of certain attributes and is a very important technology in spatial data analysis^33,34^. However, few studies have applied it to the spatial relationship analysis of different heavy metals in urban soil.

### Health risk analysis

**Figure 5:**
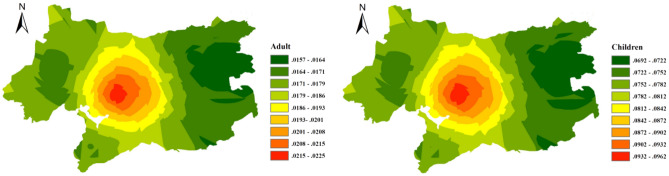
Health risk analysis of heavy metals in the urban soils of Wuxi [the figure was generated by Yan Li using the ArcGIS 10.2 (http:// https://developers.arcgis.com/)].

By using the health risk assessment model that is recommended by the U.S. EPA, this study calculated the health risks of soil heavy metals to adults and children through skin contact and ingestion. For both adults and children, the risk of soil heavy metals through ingestion was much higher than that caused by skin exposure (Table 3). For children, the total health risk that was caused by soil heavy metals is 0.078, which is four times that of adults. This may be related to children’s habits. Most children like to play with sand and climb around on the ground. These behaviours greatly increase the frequency of children contacting the soil, which thus increases the health risk caused by heavy metals in the soil. To further explore the spatial characteristics of the health risks of heavy metals in the soils of Wuxi, this study provides spatial predictions of the health risk values of soil heavy metals (Fig. 5). The total health risk values of soil heavy metals for children and adults have similar spatial distribution characteristics. High health risk values appear in the central area of Wuxi and decrease in a ring-shaped pattern. This is similar to the development degree of the city. The downtown area of Wuxi is densely populated, the pedestrian flow is very large, and the health risk of soil heavy metals in this area is very high, which poses a very serious potential threat. The health risk values for the western region of Wuxi are high, and there is also a potential threat. When compared with western Wuxi, eastern Wuxi has a lower risk.

## Conclusion

Large cities are densely populated, and heavy metals in the soil environment pose a serious threat to human health and ecological security. Affected by human activities, topography and land use types, the high concentrations of heavy metals in Wuxi are mainly concentrated in the central and western regions, and the main pollution source are considered to be coal combustion, automobile exhaust and urban garbage. The areas with high ecological risk from urban soil heavy metals in Wuxi are mainly concentrated in the middle and western parts, and the relevant management activities need to be given great attention. The high risk values for the soil heavy metal health risks of children and adults appear in the central area of Wuxi, and is consistent with the spatial distribution of population. This situation strongly increases the potential risk of soil heavy metals in Wuxi, which should receive great attention. The study clearly pointed out the sources of heavy metals in the soil of typical developed cities in China, and analyzed the risk spatial distribution characteristics of heavy metals in urban soil, which provided important information for the pollution control of heavy metals in urban soil.

## Supplementary Information

Supplementary Information.

## Data Availability

The datasets used and/or analysed during the current study are available from the corresponding author on reasonable request.
