# Supplementary material for: Study on the risk of soil heavy metal pollution in typical developed cities in eastern China
Source: Sci Rep. 2022 Mar 9;12:3855. doi: 10.1038/s41598-022-07864-3 (PMC8907225; doi:10.1038/s41598-022-07864-3)
Supplement: Supplementary file 1 — Supplementary Information. [file 41598_2022_7864_MOESM1_ESM.docx]

**Study on the risk of soil heavy metal pollution in typical developed cities in eastern China**

**Heavy metals:** 0.1 g sediment passing through a 100 mesh sieve was accurately weighed with an electronic balance and put into a polytetrafluoroethylene beaker (with a volume of 25 mL) cleaned by acid-base vibration. A small amount of Milli-Q ultrapure water was added to the beaker to wet the sample, and a pipette was used to add 2 mL of concentrated hydrochloric acid to remove the sulfide in the sample. After a short heating time, 6 mL of concentrated nitric acid was added, and the polytetrafluoroethylene beaker was placed on a heating plate at approximately 80 °C for 30 minutes. After the beaker was cooled, 5 mL of hydrofluoric acid (for the decomposition of silicate minerals, etc.) and 1 mL of perchloric acid (to analyse organic matter) were added. The solution was heated until it was clear (if the solution was still turbid, acidolysis was continued according to the above acid addition steps). The lid of the beaker was removed to allow the mixture to evaporate until it was dry and the white smoke was exhausted. Then, 2 mL of aqua regia (concentrated hydrochloric acid: concentrated nitric acid = 3:1) was added to the evaporated transparent solid weight. It was titrated to 10 mL accurately with ultrapure water, then tested and analyzed on the computer. The acidolysis methods for the blank sample and parallel sample were the same as above. The purity of acid used in the digestion process was high.

**Pb isotope:** According to the measured concentration of Pb in the sample, it was diluted to approximately 30 μg L^-1^ solution and then tested by ICP-MS, and the standard reference material (SRM981, National Institute of Standards and Technology, NIST) was used to calibrate the Pb isotope ratios tested in the study. To control the precision and accuracy of Pb isotope determination, the reference materials were measured repeatedly after every two samples were analysed. For 30 ng mL^-1^ Pb, the analytical accuracy of ^207^Pb/^206^Pb is 0.08% and that of ^208^Pb/^206^Pb is within 0.15%.

Table S1 Recoveries and detection limits for heavy metals

| **Element** | **Units** | **Detection limit** | **Average recovery (%)** |
| --- | --- | --- | --- |
| Be | mg kg^-1^ | 0.03 | 113.2 |
| Cr | mg kg^-1^ | 0.23 | 90.7 |
| Mn | mg kg^-1^ | 2.71 | 80.1 |
| Co | mg kg^-1^ | 0.07 | 79.3 |
| Ni | mg kg^-1^ | 1.01 | 84.1 |
| Cu | mg kg^-1^ | 0.06 | 76.7 |
| Zn | mg kg^-1^ | 1.78 | 91.3 |
| Cd | mg kg^-1^ | 0.01 | 81.6 |
| Pb | mg kg^-1^ | 0.17 | 85.7 |

**Health risk assessment method**

(1) Average exposure to skin contact pathways

Skin contact mainly includes human skin contact with atmospheric dust and soil. According to the US EPA human exposure risk assessment method, the skin exposure calculation model is shown in formula 1.

ADD_derm_ = $\frac{C\times SA\times CF\times SL\times ABS\times EF\times ED}{BW\times AT}$ 1

The average daily exposure of ADD_derm_ in the formula was mg kg^-1^ d^-1^; C was atmospheric dust fall or soil heavy metal element content, mg kg-1; SA was exposed skin surface area, cm^2^; SL was skin adhesion, mg cm^-2^ d^-1^; ABS was skin absorption factor, dimensionless; CF was conversion coefficient, kg mg^-1^; EF was exposure frequency, d a^-1^; ED for exposure years, a; BW for average body weight, kg; AT for average exposure time, d.

(2) Average Daily Exposure to Intake Pathway

Intake pathways mainly include human intake of wheat and rice grains, drinking tap water, soil ingestion by mistake and atmospheric dust intake. According to the US EPA human exposure risk assessment method, the intake pathway exposure calculation model is shown in formula 2.

ADD_ing_ = $\frac{C\times IngR\times CF\times EF\times ED}{BW\times AT}$ 2

In the formula, ADD_ing_ is the average daily exposure of intake pathway in mg kg^-1^ d^-1^; C is the content of heavy metal elements in atmospheric dustfall, grains, drinking water and soil, mg kg^-1^; IngR is the frequency of hand-mouth intake of dustfall or direct intake of grains, tap water and soil, mg d^-1^; other parameters represent the same significance of formula 2.

(3) Health risk characterization

The health risks of heavy metals Cd, Cr, Pb and Zn in atmospheric dustfall by different exposure routes, HQ and calculation methods of human health risks by different exposure routes and accumulation of different kinds of heavy metals, e.g. 3 - 5:

HQ_i,j_ = ADD_i,j_/RfD_i,j_ 3

HI_i_ = $\sum{HQ}_{i,j}$ 4

HI = $\sum{HI}_{i}$ 5

In the formula, HQ_ij_ and j are health risk quotients, indicating the health risk of heavy metal element i in j pathway under the same environmental medium; ADD_i,j_ are the average daily exposure amount of heavy metal element i in j pathway, in mg kg^-1^ d^-1^; RfD_i, j_ are the reference dose of daily exposure health risk of heavy metal element i in j pathway, respectively. It is mg kg^-1^ d^-1^, which indicates that every kg of heavy metals ingested by human body every day will not cause the maximum amount of pollutants that cause adverse reactions to human body; HI_i_ is the total health risk caused by heavy metals i in the same environmental medium; HI is the total health risk caused by accumulation of heavy metals in multiple ways in the same environmental medium. When HQ and HI are less than or equal to 1.0, it is considered that the health risk of heavy metals in the study area is less or no obvious harm to human body; when HQ and HI are greater than 1.0, it is considered that heavy metals in the study area may cause harm to human body. Table. S2 lists the reference doses of heavy metals in different exposure routes for human health risk, RfD.

| Table. S2 Meaning and value of calculation parameters for daily average exposure of heavy metals | | | | | |
| --- | --- | --- | --- | --- | --- |
| Items | Abbreviation | Meaning | Unit | Children's value | Adult value |
| Basic parameters | C | Heavy metal concentration | mg kg-1 |  |  |
|  | BW | Average weight | kg | 17.6 | 67.6 |
| Exposure behavior parameters | EF | Exposure frequency | d a-1 | 180 | 180 |
|  | ED | Exposure period | a | 6 | 24 |
|  | AT | Average exposure time | d | 365×ED | 365×ED |
|  | BW | Average weight | kg | 17.6 | 67.6 |
|  | CF | Unit conversion | kg mg-1 | 1×10-6 | 1×10-6 |
| Hand-mouth feeding | IngR | Feeding dust reduction rate | mg d-1 | 200 | 100 |
| Respiratory intake | InhR | Respiratory rate | m3 d-1 | 7.63 | 20 |
|  | PEF | Particulate emission factor | mg3 kg-1 | 1.32×109 | 1.32×109 |
| Skin contact | SL | Skin adhesion | mg cm-2 | 0.2 | 0.07 |
|  | SA | Exposure of skin area | cm2 d-1 | 1077.5 | 2011.25 |
|  | ABS | Skin absorption factor |  | 0.001 | 0.001 |

**Table S3. Pb isotopic composition of known anthropogenic and natural sources**

| Category | Source | ^208^Pb/^206^Pb | ^206^Pb/^207^Pb | Reference |
| --- | --- | --- | --- | --- |
| Anthropogenic sources | Automotive exhaust | 2.103 | 1.17 | Gao et al., 2004 ^1^ |
|  | Automotive exhaust | 2.112 | 1.161 | Gao et al., 2004 ^1^ |
|  | Coal combustion | 2.111 | 1.163 | Tan et al., 2006 ^2^ |
|  | Municipal wastes | 2.101 | 1.169 | Hu et al., 2013 ^3^ |
|  | Municipal wastes | 2.095 | 1.174 | Hu et al., 2013 ^3^ |
|  | Factory | 2.109 | 1.158 | Hu et al., 2013 ^3^ |
|  | Factory | 2.084 | 1.18 | Hu et al., 2013 ^3^ |
|  | Factory | 2.112 | 1.16 | Hu et al., 2013 ^3^ |
| Natural sources | Uncontaminated soils and Granite | 2.077 | 1.195 | Zhu et al., 2001 ^4^ |
|  | Uncontaminated soils and Granite | 2.086 | 1.183 | Zhu et al., 2001 ^4^ |
|  | Uncontaminated soils and Granite | 2.096 | 1.184 | Zhu et al., 2001 ^4^ |
|  | Uncontaminated soils and Granite | 2.089 | 1.193 | Hu et al., 2013 ^3^ |
|  | Uncontaminated soils and Granite | 2.061 | 1.214 | Hu et al., 2013 ^3^ |

**Reference**

1, Gao, Z.Y., Yin, G., Ni, S.J., Zhang, C.J., 2004. Geochemical feature of the urban environmental lead isotope in Chendu city. Carsologica Sinica.

2, Tan, M.G., Zhang, G.L., Li, X.L., Zhang, Y.X., Yue, W.S., Chen, J.M., Wang, Y.S., Li, A.G., Li, Y., Zhang, Y.M., 2006. Comprehensive study of lead pollution in Shanghai by multiple techniques. Analytical Chemistry 78, 8044.

3, Hu, G., Yu, R., Zheng, Z., 2013. Application of stable lead isotopes in tracing heavy-metal pollution sources in the sediments. Acta Scientiae Circumstantiae 33, 1326-1331.

4, Zhu, B.Q., Chen, Y.W., Peng, J.H., 2001. Lead isotope geochemistry of the urban environment in the Pearl River Delta. Applied Geochemistry 16, 409-417.
